# Supplementary material for: Adaptive Therapy for Metastatic Melanoma: Predictions from Patient Calibrated Mathematical Models
Source: Cancers (Basel). 2021 Feb 16;13(4):823. doi: 10.3390/cancers13040823 (PMC7920057; doi:10.3390/cancers13040823)
Supplement: Supplementary file 1 [file cancers-13-00823-s001.zip › Cancers-1091112_supplementary_conversion/cancers-1091112-supplementary.docx]

Supplementary Material: Adaptive Therapy for Metastatic Melanoma: Predictions from Patient Calibrated Mathematical Models

Eunjung Kim ^1,^ *, Joel S. Brown ^2^, Zeynep Eroglu^3^ and Alexander R.A. Anderson ^2,^ *


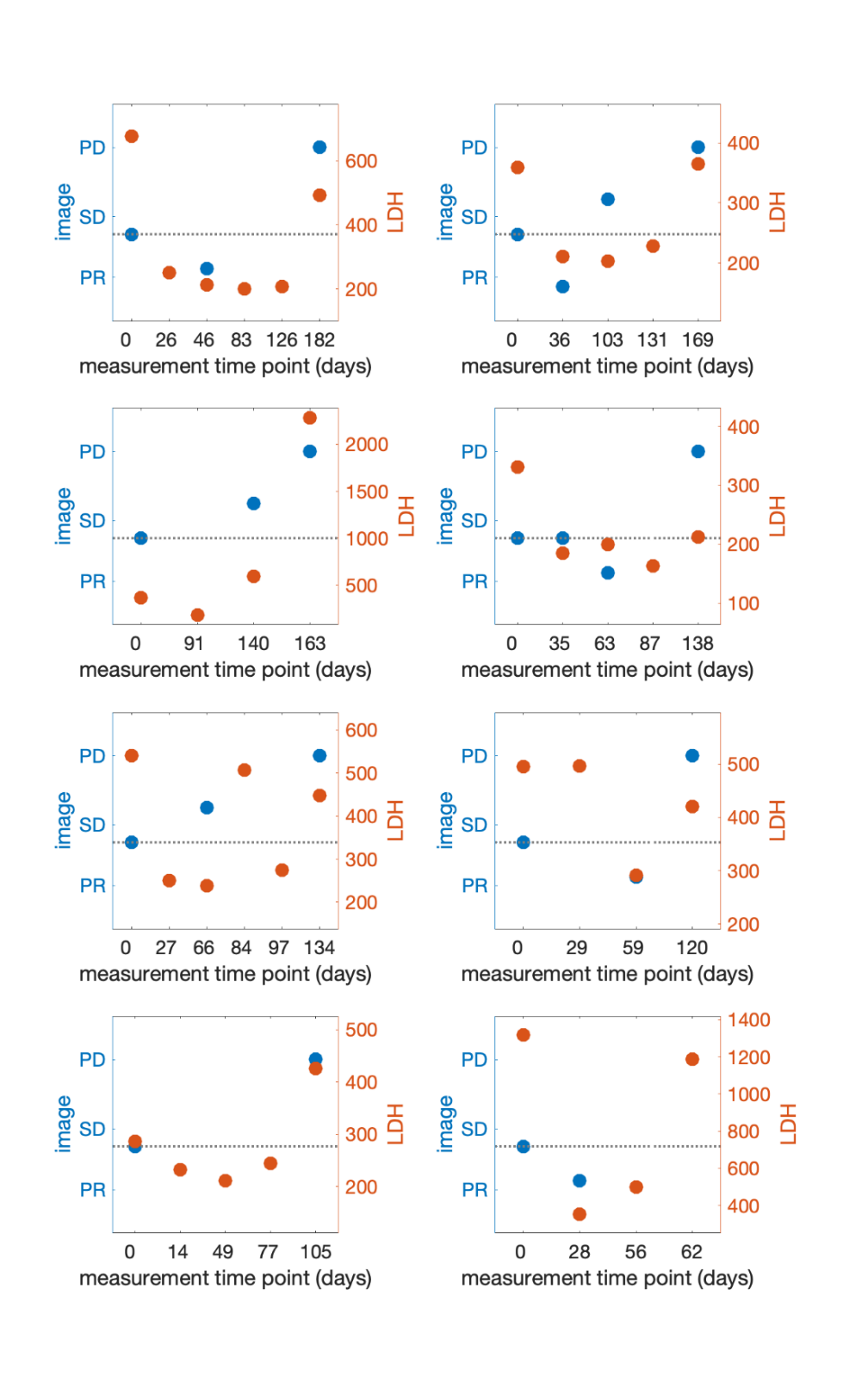


**Figure S1.** Patient LDH and image (CT) data. Partial Response (PR): more than 30% reduction in the sum of target tumor diameters, Stable Disease (SD): up to 20% increase in the sum of target tumor diameters, Progressive Disease (PD): more than 20% increase in the sum of target tumor diameters. Blue: CT image (left axis), Orange: LDH (right y-axis).

**Table S1.** Patient LDH (yellow) and response evaluation data (blue). T0-T5: measurement time.

|  | **T0** | **T1** | **T2** | **T3** | **T4** | **T5** | **Time to Progression** |
| --- | --- | --- | --- | --- | --- | --- | --- |
| **P1** | 677 | 251 | 212(PR) | 200 | 208 | 493(PD) | 26 weeks |
| **P2** | 359 | 211(PR) | 203(SD) | 228 | 364(PD) |  | 24 weeks |
| **P3** | 366 | 181 | 598(SD) | 2285(PD) |  |  | 23 weeks |
| **P4** | 331 | 185 | 200(PR) | 164 | 213 (PD) |  | 20 weeks |
| **P5** | 541 | 250 | 238(SD) | 507 | 275 | 448(PD) | 19 weeks |
| **P6** | 496 | 497 | 291(PR) | 421(PD) |  |  | 17 weeks |
| **P7** | 286 | 232 | 211 | 244 | 426(PD) |  | 15 weeks |
| **P8** | 1320 | 353(PR) | 501 | 1188(PD) |  |  | 9 weeks |


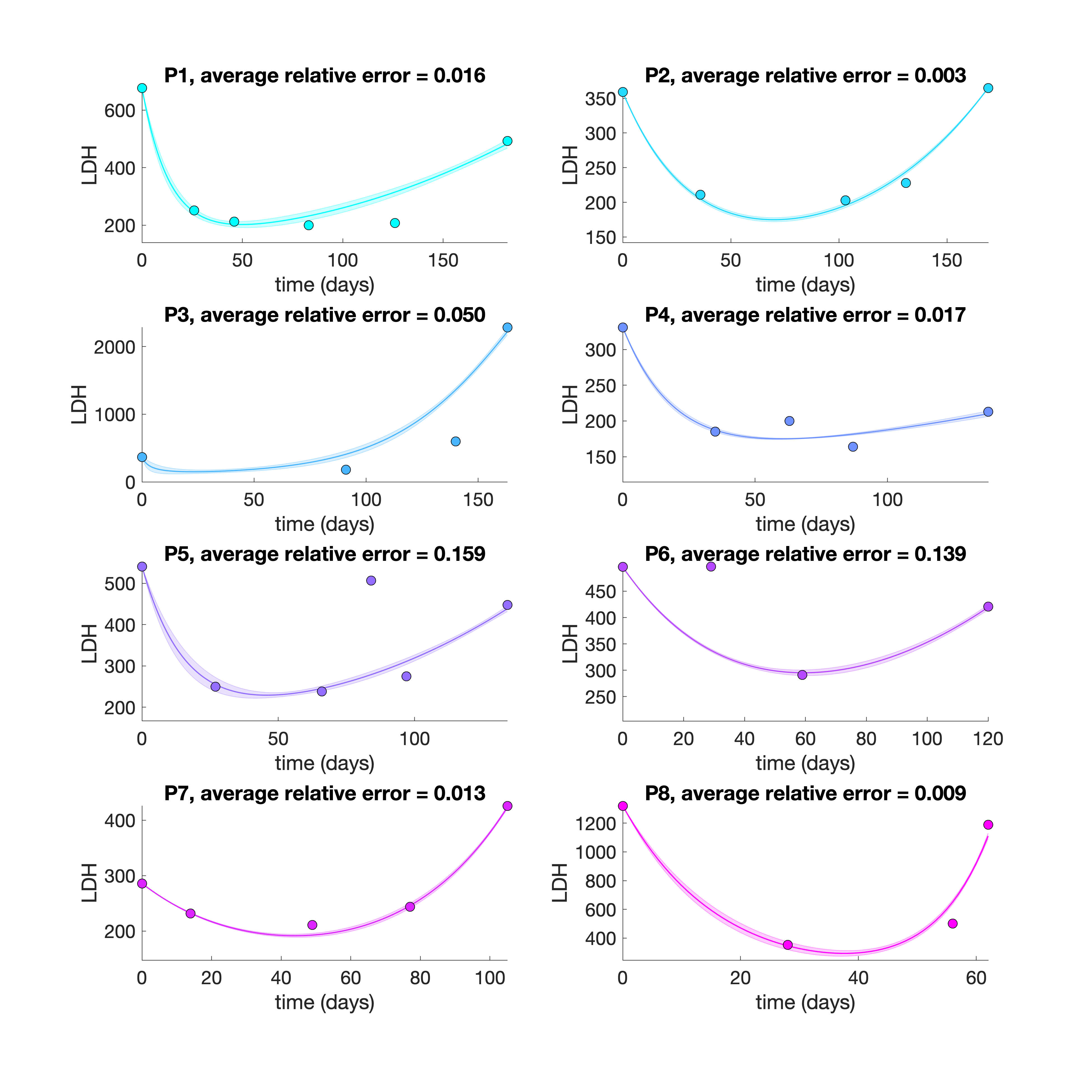


**Figure S2.** LV model calibration. We estimated cell growth rates, carrying capacity, drug-sensitive population death rate, and competition coefficient. The model parameters were estimated by a method described in the Parameter Estimation section. In brief, we estimated model parameters that minimized the difference between model predicted LDH and each patient LDH. The implicit filtering algorithm with different initial values was used to identify various sets of parameters that equally fit to patient data. We selected the top 50 best-matched parameters to generate fitted curves. Thick line: mean of model predictions, shadow: standard deviation, dot: patient LDH measurement.


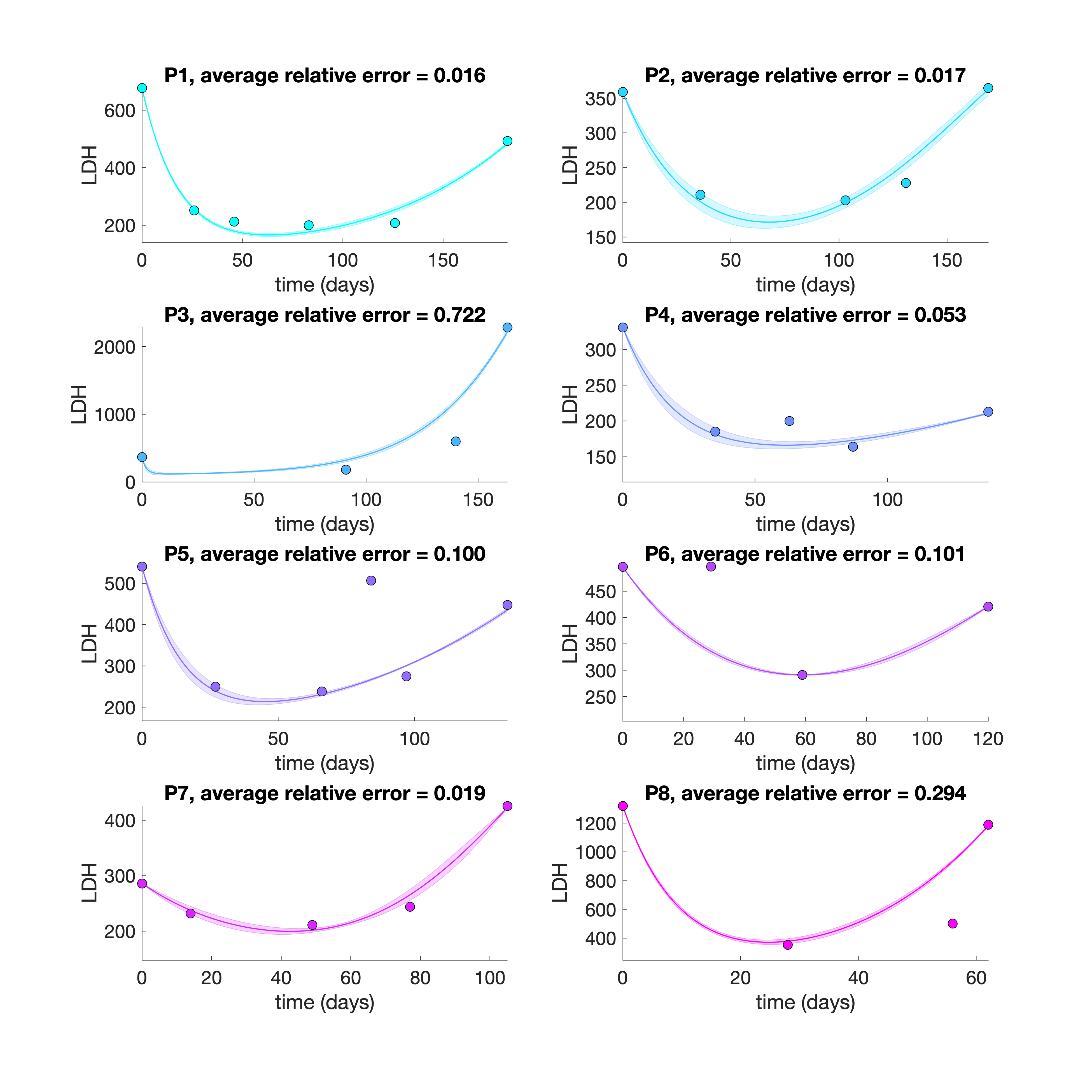


**Figure S3.** SW model calibration. For SW model, we estimated growth rates, carrying capacity, and transition rates. Thick line: mean value of model predictions, shadow: standard deviation, dot: patient LDH data.


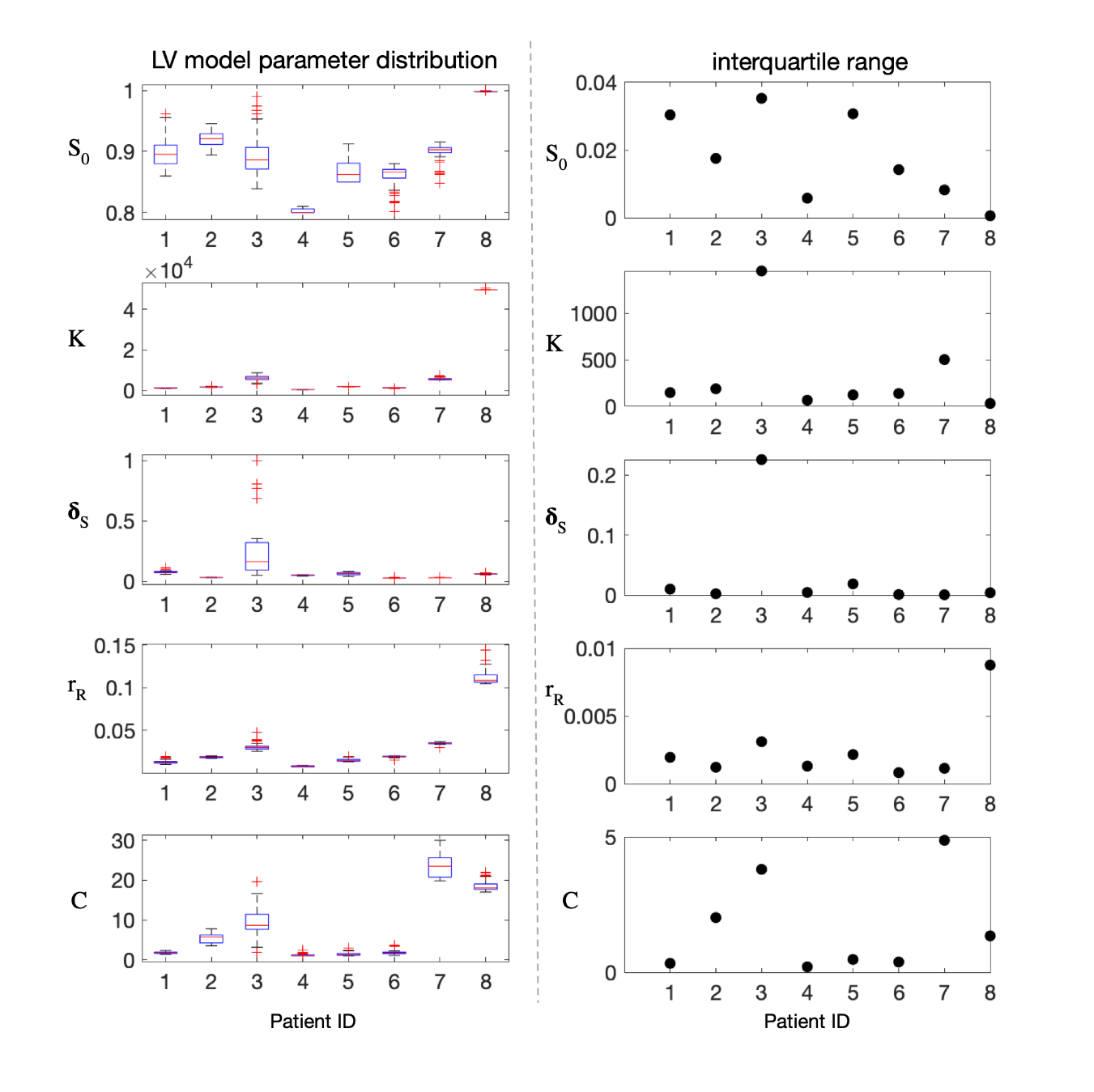


**Figure S4.** LV model parameter variability assessment. Left: distributions of LV model parameters for all the 8 patients. Right: Interquartile range of each set of estimated parameters.


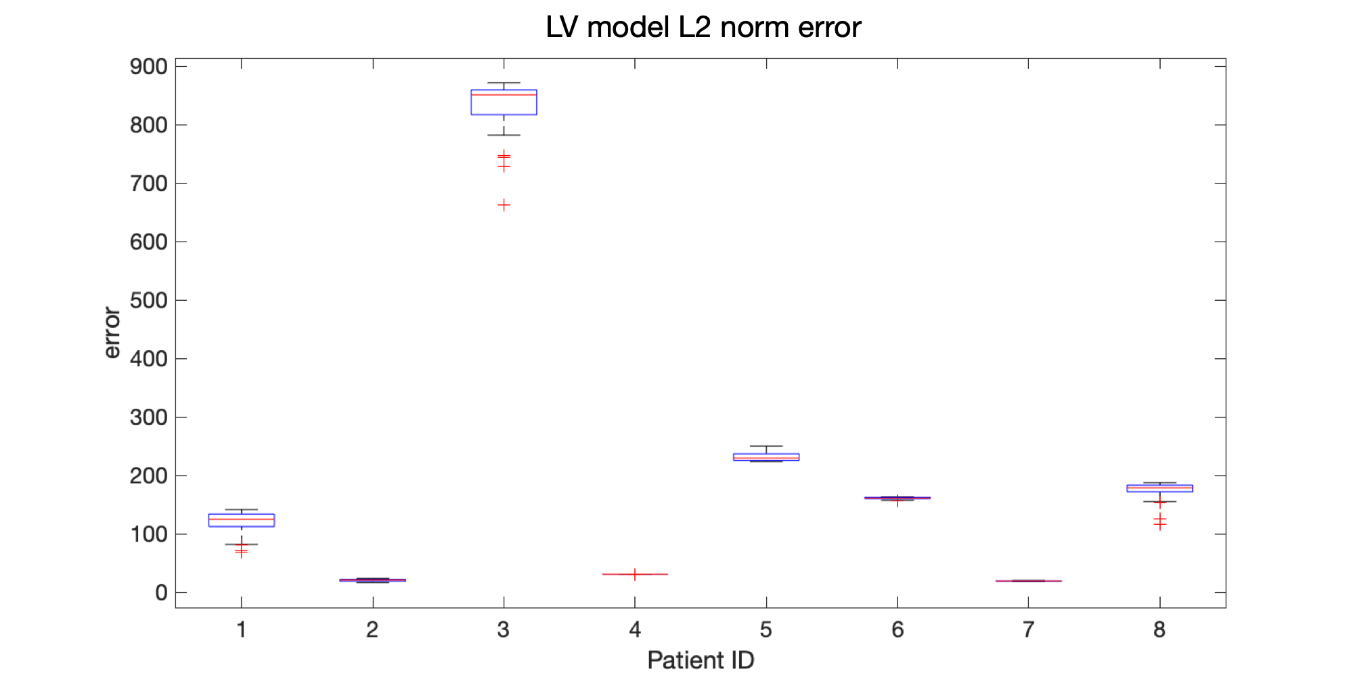


**Figure S5.** L_2_ norm of LV model calibration errors. For each patient, we calculated an L2 norm error ($\boldsymbol{E}\left( \boldsymbol{i} \right)=\sqrt{\sum_{\boldsymbol{j}=\boldsymbol{1}}^{\boldsymbol{n}} {(\boldsymbol{M}(\boldsymbol{t}_{\boldsymbol{j}})-\boldsymbol{D}(\boldsymbol{t}_{\boldsymbol{j}}))}^{\boldsymbol{2}},} \boldsymbol{i}=\boldsymbol{1},\boldsymbol{2},\ldots,\boldsymbol{50}$, where $\boldsymbol{M}(\boldsymbol{t}_{\boldsymbol{j}})$ is a model prediction at time $\boldsymbol{t}_{\boldsymbol{j}}$ and $\boldsymbol{D}\left( \boldsymbol{t}_{\boldsymbol{j}} \right)$is a data value at time $\boldsymbol{t}_{\boldsymbol{j}}$). The distributions of the errors are presented. X-axis: patient ID & y-axis: error.


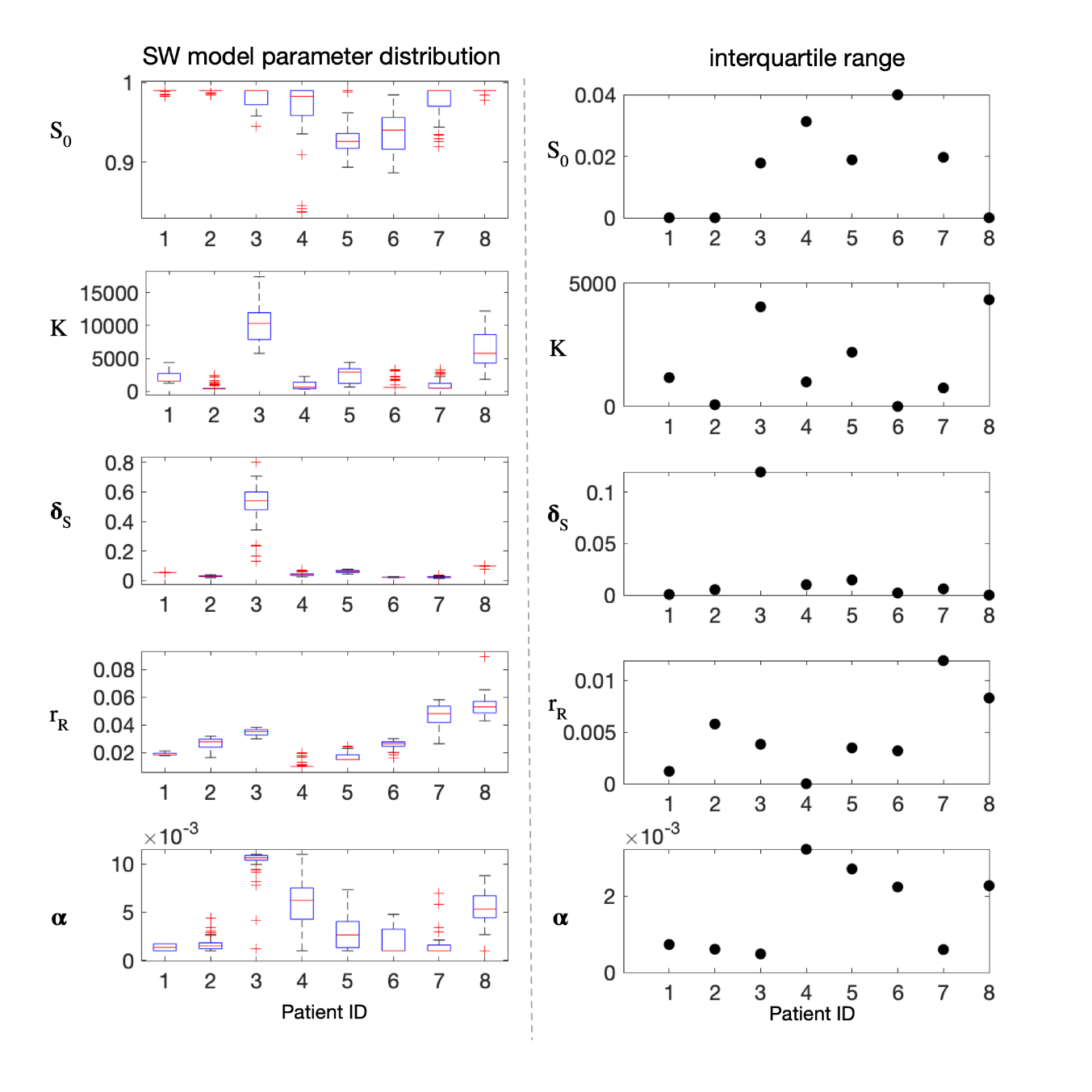


**Figure S6.** SW model parameter variability assessment. Left: distributions of LV model parameters for all the 8 patients. Right: Interquartile range of each set of estimated parameters.


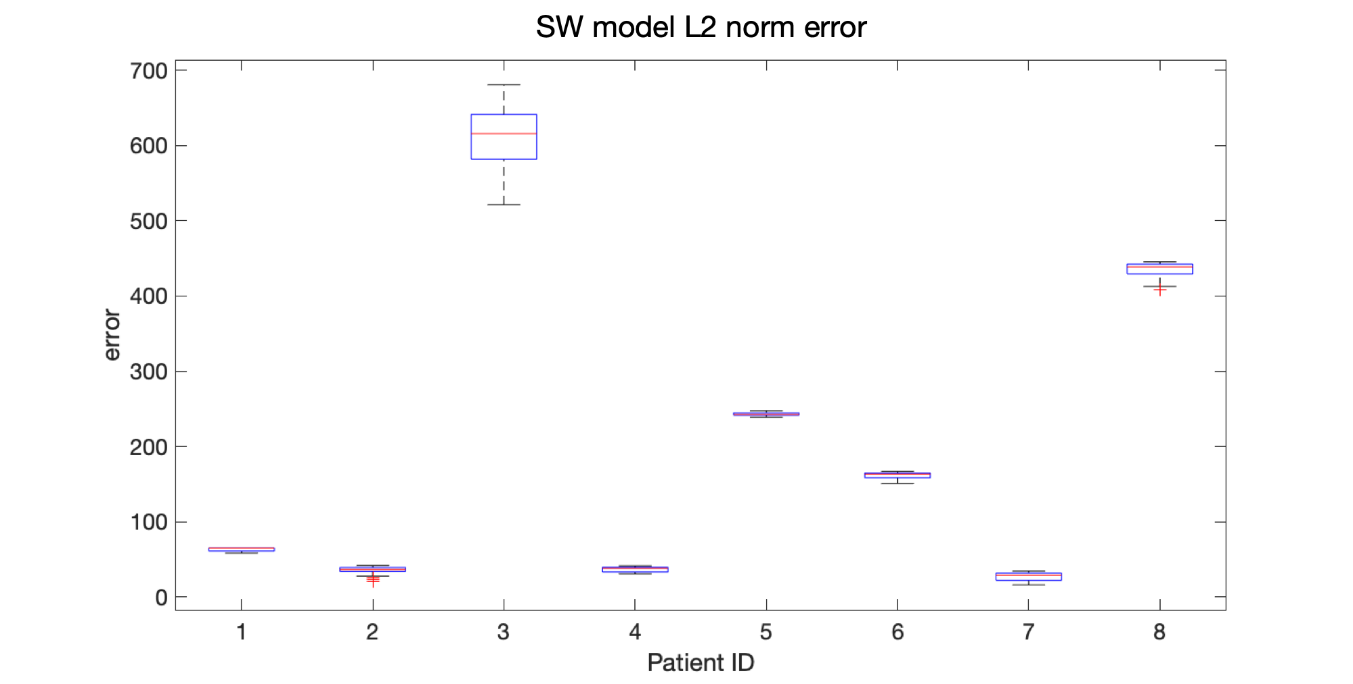


**Figure S7.** L_2_ norm of SW model calibration errors. For each patient, we calculated an L2 norm error ($\boldsymbol{E}\left( \boldsymbol{i} \right)=\sqrt{\sum_{\boldsymbol{j}=\boldsymbol{1}}^{\boldsymbol{n}} {(\boldsymbol{M}(\boldsymbol{t}_{\boldsymbol{j}})-\boldsymbol{D}(\boldsymbol{t}_{\boldsymbol{j}}))}^{\boldsymbol{2}},} \boldsymbol{i}=\boldsymbol{1},\boldsymbol{2},\ldots,\boldsymbol{50}$, where $\boldsymbol{M}(\boldsymbol{t}_{\boldsymbol{j}})$ is a model prediction at time $\boldsymbol{t}_{\boldsymbol{j}}$ and $\boldsymbol{D}\left( \boldsymbol{t}_{\boldsymbol{j}} \right)$is a data value at time $\boldsymbol{t}_{\boldsymbol{j}}$). The distributions of the errors are presented. X-axis: patient ID & y-axis: error.


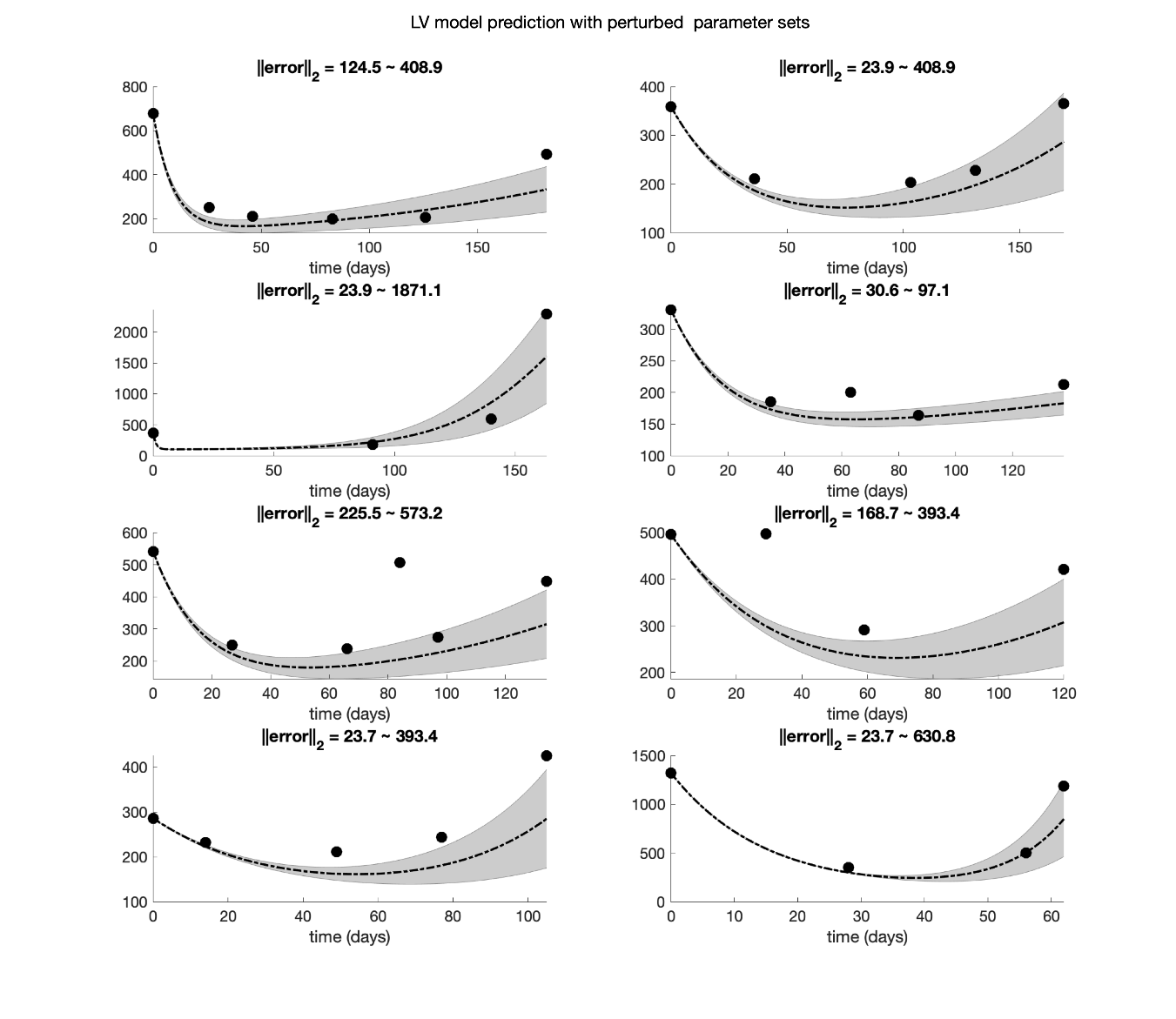


**Figure S8.** LV model predictions with perturbed parameters. Among the 50 estimated parameter sets, we chose the one that produced the largest error and perturbed each parameter by 10%. Then, we simulated continuous therapy with the perturbed parameter set. We repeat this over 100 times to check if the perturbed parameter (not optimal one) can also explain the observed patient data. Dots: patient data points, dashed line: mean value of LV model predictions, shade: standard deviation of model predictions.


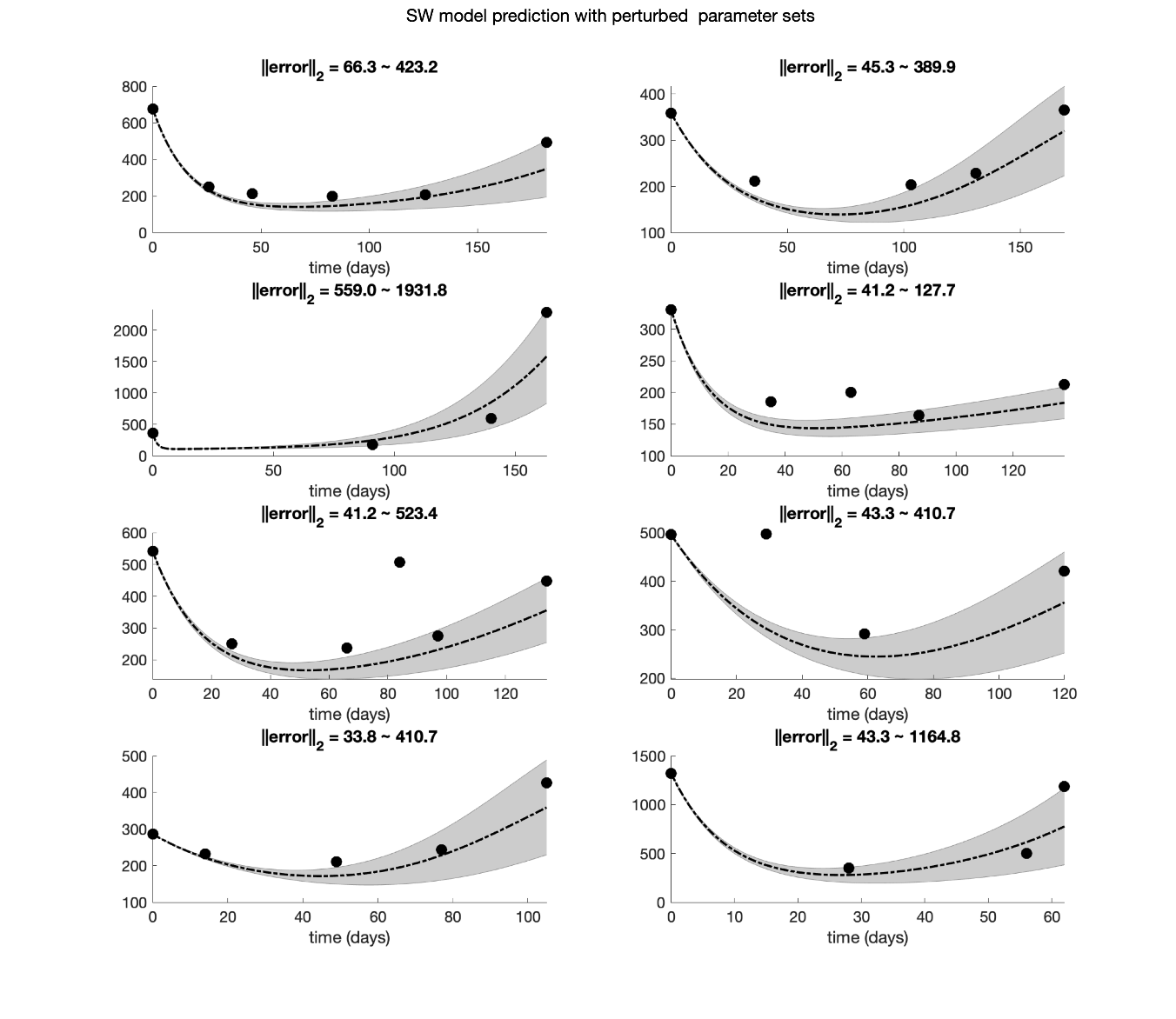


**Figure S9.** SW model predictions with perturbed parameters. Among the 50 estimated parameter sets of the SW model, we chose the one that produced the largest error and perturbed each parameter by 10%. Then, we simulated continuous therapy with the perturbed parameter set. We repeat this over 100 times to check if the perturbed parameter (not optimal one) can also explain the observed patient data. Dots: patient data points, dashed line: mean value of SW model predictions, shade: standard deviation of model predictions.


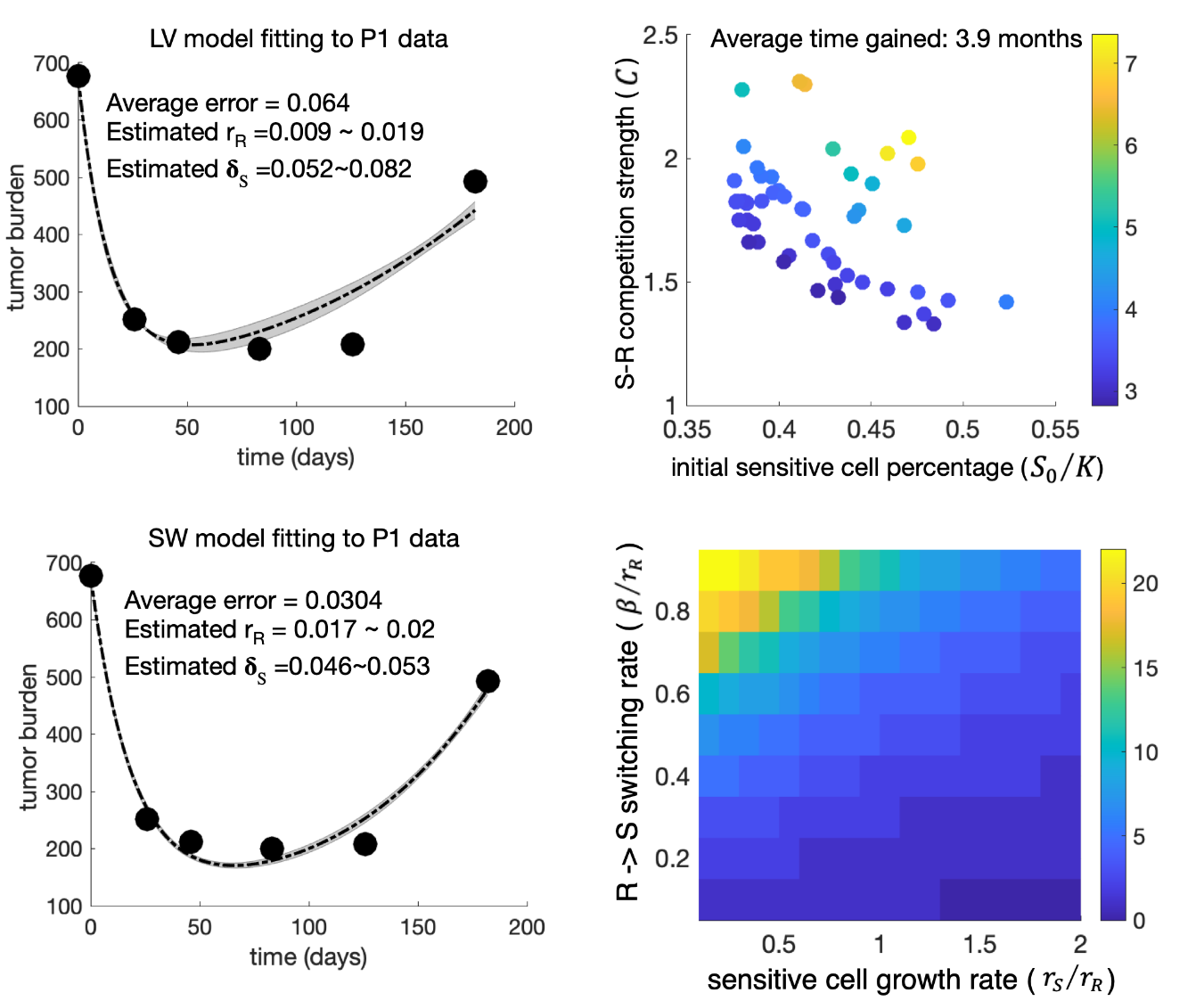


**Figure S10.** Model fitting to the patient 1 data. The model parameters ($r_{R},\delta_{S}$)were re-estimated while the others were fixed as previously. With the new parameters, we simulated an adaptive therapy with various choices of $r_{S}$ and $\beta.$ Upper panel: LV model fits to the P1 data and adaptive therapy simulation. The average time gained for this patient is 3.9 months (range 3~7.2 months). The results are similar to the predictions with the previous model parameters (Figure 4B, P1). Bottom panel: SW model fits to the data. SW model predicted time gained (0~22 months) for the patient P1. The results are similar to previous predictions (Figure 4D).


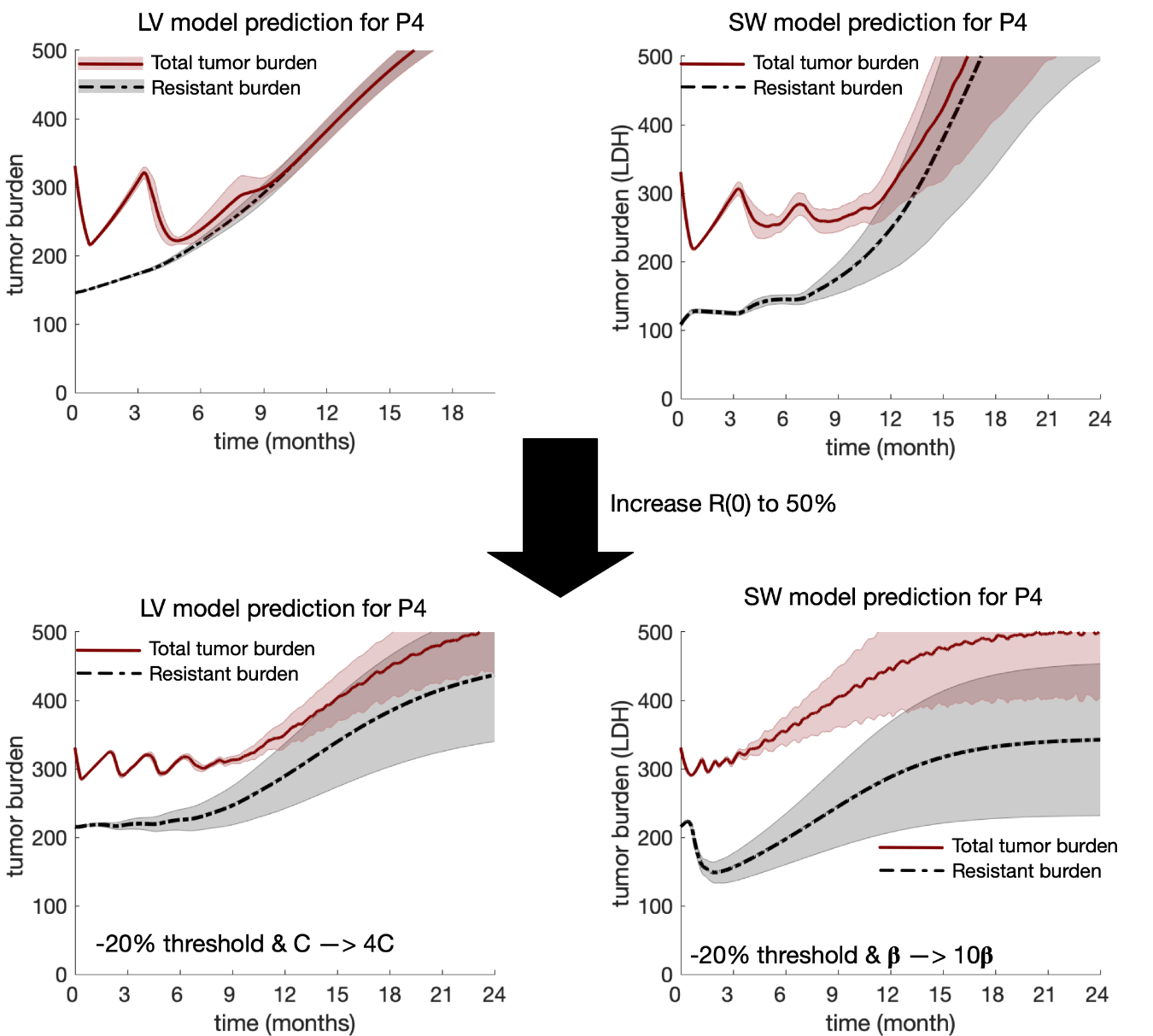


**Figure S11.** Model projection of drug-resistant population (*R*). Among the 8 patients, we selected patient 4 as a representative patient because the *R* (0) population for the patient was estimated to be the largest (~20% vs. ~10% others). The upper panel shows LV and SW model projections for the *R* population. Then the *R* (0) was increased to 50% of the initial total population. The LV model predicted an adaptive therapy could maintain tumor volume up to 12 months if we increased the competition coefficient C to four times (C🡪4C) and used a -20% threshold for the treatment stop (bottom left panel). The SW model predicted an adaptive therapy with a treatment stop threshold of -20% could maintain tumor burden up to 15 months if the transition rate from *R* to *S* was increased to 10 times ($\beta\to10\beta$). Thick line: mean value of model predicted total burden, dashed line: mean value of model predicted R population, shadow: standard deviation.

| 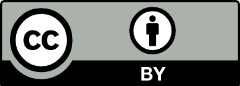 | © 2020 by the authors. Submitted for possible open access publication under the terms and conditions of the Creative Commons Attribution (CC BY) license (http://creativecommons.org/licenses/by/4.0/). |
| --- | --- |
